# Relato de Caso de Doença Cardíaca Valvular Complicada com um Cisto Sanguíneo no Átrio Direito. Revisão da Literatura

**DOI:** 10.36660/abc.20210063

**Published:** 2021-11-22

**Authors:** Shu Jiang, Xiao-Cong Wang, Yan-Li Zhang, Wei Yu, Li-Ping Pei, Yan Ma

**Affiliations:** 1 Jilin University First Hospital Changchun China Jilin University First Hospital, Changchun – China

**Keywords:** Cistos Sanguíneos/genética, Cistos /cirurgia, Doença das Valvas Cardíacas, Átrio do Coração/fisiologia, Função Atrial, Ecocardiografia/métodos

## Introdução

Os cistos sanguíneos no coração são frequentemente reconhecidos como benignos. Geralmente não causam sintomas clínicos e são frequentemente encontrados em bebês menores de dois meses.^1^ Na maioria dos casos, os cistos sanguíneos estão nas válvulas cardíacas ou suas estruturas de suporte.^2^ Os cistos cardíacos em adultos são extremamente raros, especialmente nas câmaras do coração. Aqui, relatamos um caso de doença cardíaca valvular complicada com um cisto sanguíneo no átrio direito. As ecocardiografias transtorácica, de contraste e esofágica foram utilizadas para examinar o paciente.

## Relato de caso

A paciente era uma mulher de 64 anos com palpitações e falta de ar após o exercício por quatro meses. Nas duas semanas anteriores, os sintomas haviam piorado. Os hospitais locais recomendaram encaminhamento para hospitais superiores, e assim foi encaminhada ao nosso hospital para tratamento. Ela tinha um histórico de hipertensão por sete anos, fumante durante 30 anos e cirurgia de histeromisma há mais de 20 anos. Não tinha histórico de diabetes e negava tuberculose, hepatite e outras doenças infecciosas. O paciente não tinha um registro claro de medicação. Na admissão, seus sinais vitais estavam estáveis. O eletrocardiograma mostrou fibrilação atrial. Murmúrios sistólicos puderam ser ouvidos na área de auscultação da válvula mitral. Não houve murmúrios em outras áreas de auscultação de válvulas.

**Figura 1:**
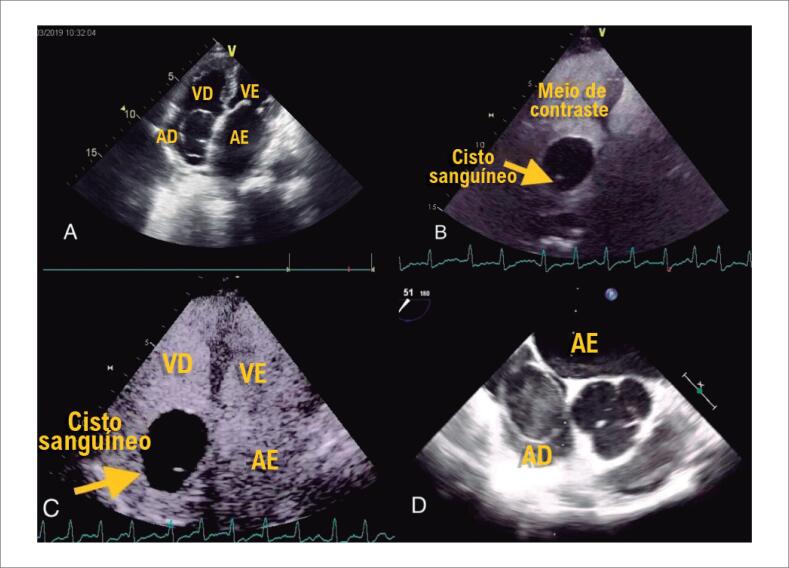
A) A ecocardiografia transtorácica mostrou lesão anecóica circular no átrio direito. B, C) A imagem de perfusão de contraste mostrou uma estrutura cística com septo e ausência de perfusão na massa. D) A ecocardiografia transesofágica intraoperatória revelou um eco circular no átrio direito, com contraste ecocardiográfico espontâneo e septação. VD: ventrículo direito; VE: ventrículo esquerdo; AD: átrio direito; AE: átrio esquerdo.

A ecocardiografia transtorácica revelou átrios aumentados bilateralmente, estenose mitral leve e insuficiência grave, com função sistólica ventricular esquerda normal (FEVE = 52%). Além disso, tinha uma lesão anecóica circular de aproximadamente 30x41 mm de tamanho no átrio direito, que não causava obstrução da válvula tricúspide (Figura 1A).

Um dispositivo de diagnóstico de ultrassom GE Vivid E9 com uma sonda M5sC foi usado para uma ecocardiografia de contraste examinação. SonoVue (1 ml) foi injetado lentamente através da veia cubital mediana durante 1 minuto. O artefato de cintilação foi acionado aproximadamente 2 minutos após a injeção do agente de contraste. As microbolhas no miocárdio foram destruídas rapidamente quando um pulso de alta IM foi emitido. Em seguida, o processo de enchimento do agente de contraste no miocárdio e tumores foi observado em um estado de baixa IM. A imagem de perfusão contrastada mostrou estrutura cística com septo de aproximadamente 36x42 mm no átrio direito e ausência de perfusão na massa. (Figura 1B, C).

Ecocardiografia transesofágica intraoperatória revelou eco circular em átrio direito, com contraste ecocardiográfico espontâneo e septação, o que consideramos cisto sanguíneo (Figure 1D).

**Figura 2:**
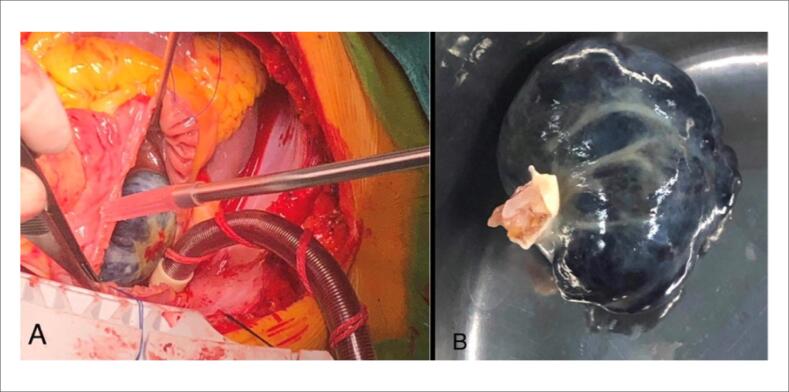
A, B) Achados cirúrgicos. O tumor estava localizado no átrio direito ligado à fossa oval, perto do orifício da veia cava inferior, por um pedículo de aproximadamente 5 mm de diâmetro.

O paciente foi submetido à excisão de massa atrial direita, substituição da válvula mitral e valvuloplastia tricúspide. A massa estava localizada no átrio direito ligado à fossa oval, perto do orifício da veia cava inferior, por um pedículo de aproximadamente 5 mm de diâmetro. Tinha cerca de 40 x 40 mm de tamanho (Figura 2A, B). A cápsula estava intacta, dura e roxa-preta. O tecido septo atrial conectado ao tumor e ao pedículo foi completamente removido. Foi realizada substituição da válvula mitral e valvuloplastia tricúspide.

**Figura 3:**
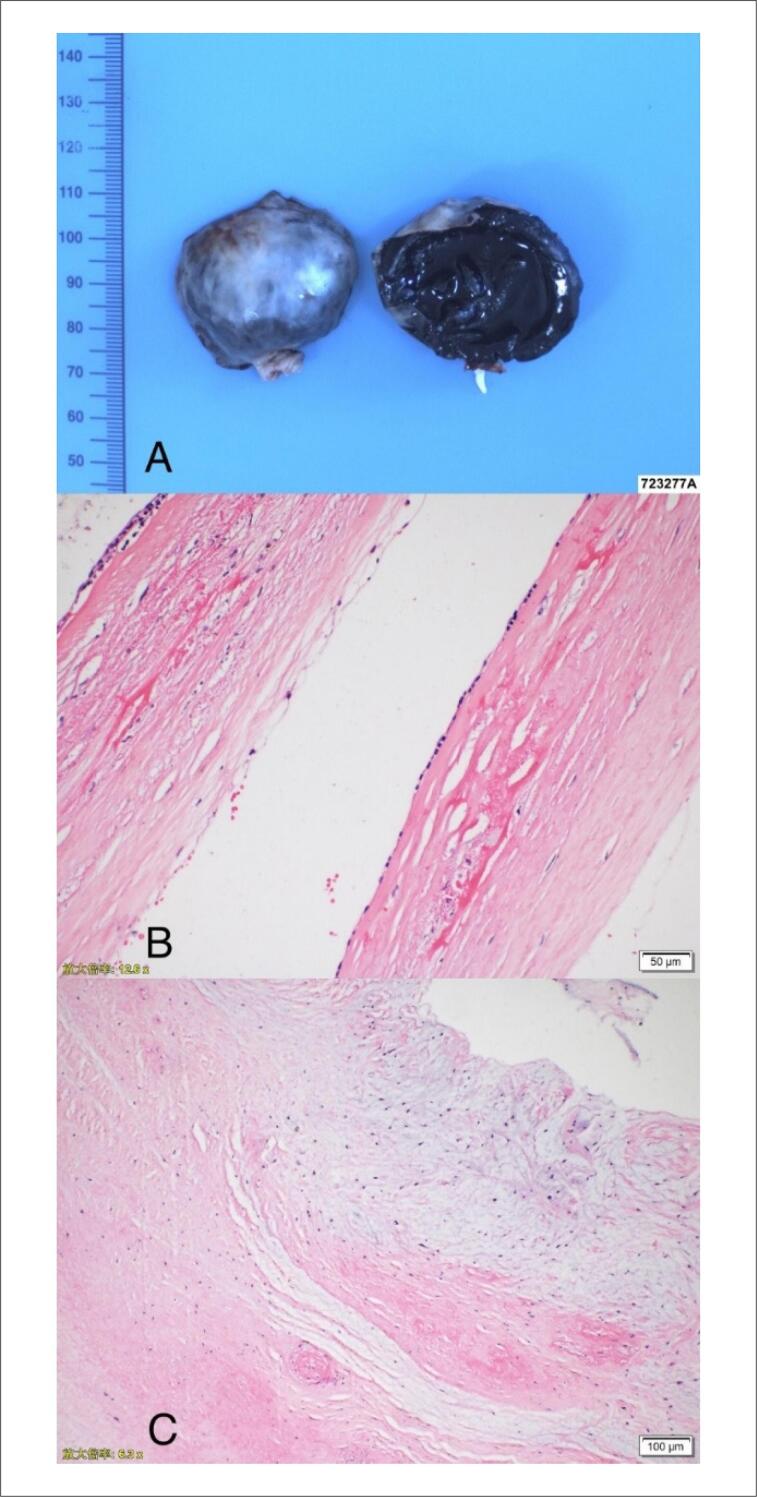
A, B) Achados patológicos. A massa foi preenchida com tecido fibrocístico semelhante à parede, hiperplasia de tecido fibroso intersticial, degeneração de hialina e degeneração mucinosa na massa do átrio direito, e tecido de coagulação maciça. C: O tecido da válvula examinado mostrou degeneração de hialina e degeneração mucinosa com infiltração dispersa de células inflamatórias crônicas.

O exame macroscópico mostrou que a massa do átrio direito era redonda com um volume de 45x35x27 mm, tinha uma cápsula completa e um pedículo estreito na superfície. A seção de massa era unilocular, a parede cística tinha 1-2 mm de espessura, o revestimento interno era liso, e o conteúdo eram principalmente coagulos. O exame microscópico de luz de seções embebidas em parafina mostrou tecido semelhante a parede fibrocística, hiperplasia de tecido fibroso intersticial, degeneração hialina, degeneração mucinosa na massa do átrio direito e tecido de coagulação maciço, o que estava de acordo com as alterações do cisto (Figura 3A, B). O tecido da válvula examinado mostrou degeneração de hialina e degeneração mucinosa com infiltração dispersa de células inflamatórias crônicas (Figura 3C).

O paciente se recuperou bem após a operação. Nenhuma massa anormal foi encontrada no átrio direito por ecocardiografia. Além disso, sua prótese de válvula mitral e a função da válvula tricúspide estavam normais.

## Discussão

Elsasser relatou pela primeira vez cistos sanguíneos em 1844.^3^ Geralmente são de origem congênita e são encontrados poucos meses após o nascimento, desaparecendo espontaneamente com o tempo. Eles ocorrem principalmente em válvulas cardíacas, como válvulas mitral, tricúspide, aórtica e válvulas pulmonares. Às vezes, eles existem no átrio esquerdo e átrio direito.^4,5^ Cistos sanguíneos em adultos são extremamente raros, especialmente na cavidade cardíaca. Existem várias hipóteses sobre as causas da formação do hemocisto: 1. Durante o desenvolvimento das válvulas, cistos de sangue são formados porque o sangue é espremido e fica preso na lacuna que posteriormente se fecha. 2. Possíveis alterações heteroplásicas no tecido mesotelial pericárdico primário. 3. Sakakibara et al.,^6^ sugerem que um bloqueio repentino da circulação causa cistos sanguíneos no átrio ou ventrículo. Eles acreditavam que as tendências de hipóxia, inflamação e sangramento poderiam ser responsáveis pela transformação do hematoma endocárdico em hemocistos. 4. Os cistos sanguíneos representam apenas vasos sanguíneos dilatados. 5. Degeneração mucinosa.^7^ No entanto, ainda não há consenso sobre a causa dos cistos sanguíneos.^4,8,9^ A paciente atual tinha insuficiência mitral. O exame patológico da válvula mostrou degeneração de hialina e degeneração mucinosaa. Presume-se que isso tenha relação com a formação de cistos sanguíneos.

Os tumores cardíacos são principalmente benignos e mixomatosos. Eles podem ocorrer em qualquer idade. O mixoma geralmente ocorre na superfície do endocárdio da cavidade cardíaca, 60% -80% das vezes no átrio esquerdo. O pedículo se prende à fossa oval do septo atrial e tem uma certa amplitude de movimento com o ciclo cardíaco.

No entanto, o mixoma é geralmente uma massa de eco forte redondo ou oval com ecos internos regulares. Se houver necrose no centro, pode ser uma área anecóica.

A ecocardiografia de contraste do ventrículo esquerdo de mixoma mostra que o meio de contraste é esparso, mas realçado na lesão. Sua intensidade é inferior à do tecido miocárdico adjacente, o que não condiz com o caso.

As manifestações ecocardiográficas da equinococose cardíaca são lesões císticas que ocupam espaço no coração. A distribuição de cistos no coração está relacionada principalmente com o suprimento de sangue do miocárdio. Portanto, o local mais comum é o miocárdio da parede ventricular, que tem o maior suprimento de sangue do coração, seguido pela parede atrial, e a cavidade cardíaca é menos frequentemente envolvida.^10^ No entanto, o paciente não tinha uma história clara de contato com bovinos ou ovinos ou uma dieta suja, de modo que o diagnóstico não foi apoiado.

De 1958 a 2020, havia apenas dez artigos sobre cistos sanguíneos no átrio direito confirmados por patologia. Nossa revisão da literatura em língua inglesa revelou dez outras entradas desde 1996, descrevendo um total de 10 pacientes com detalhes desses cistos sanguíneos no átrio direito (Tabela 1). Os sintomas dos cistos sanguíneos relatados variam. Quatro pacientes eram assintomáticos.^11–14^ Outros pacientes apresentaram sintomas. Os sintomas comuns foram dispneia e desconforto no peito, um caso de dor crônica e um de dor de cabeça. O local de apresentação também foi diferente. Seis cistos foram no septo atrial,^11,13-17^ 2 na fossa oval,^12,18^ 1 na válvula tricúspide^19^ e 1 na válvula sinusal coronária.^20^ A calcificação foi encontrada em 5 dos cistos sanguíneos.^12,14-16,18^ Após a cirurgia, a maioria dos pacientes se recuperou1,^11-15,18,20^ 2 pacientes não tiveram um prognóstico explicado^16,19^ e 1 paciente morreu.^17^

**Tabela 1:** Resumo clínico dos casos de cistos sanguíneos encontrados na literatura inglesa desde 1958

Paciente	Primeiro autor	Ano	Gênero	Idade	Tamanho do tumor	Local	Cisto sanguíneo com cálcio	Sintoma	Histórico	Prognóstico
1	H Niinam^15^	1996	F	59	20x20mm	septo atrial entre o fossa ovalis e tricúsulide válvula	Sim	episódios periódicos de pressão substernal e tosse seca	nenhum	Recuperação
2	Hiroyuki Tanaka^11^	2003	M	52	40x30mm	septo atrial entre o fossa ovalis e válvula tricúspide	Não	nenhum	câncer gástrico	Recuperação
3	Gernot Seebacher^18^	2006	F	65	44x20mm	fossa ovalis de septo atrial	Sim	dispneia, angina e taquicardia	nenhum	Recuperação
4	Kaoru Otsuka^12^	2007	M	56	30x20mm	fossa ovalis do septo atrial	Sim	nenhum	nenhum	Recuperação
5	Tomasa Centella^16^	2015	Desconhecido	62	28x28mm	septo atrial	Sim	dor de cabeça	ferritina elevada não relacionada ao gene de hemocromatose, uma hérnia de hiato, esofagite leve e cistos de seio renal	Desconhecido
6	Hiroyuki Otsuka^13^	2016	F	85	30x30mm, 25x25mm	septo atrial entre o fossa ovalis ea válvula tricúspide	Não	nenhum	síndrome do nódulo sinusal	Recuperação
7	Hilary Bews^19^	2018	F	62	45x54mm	válvula tricúspide	Não	dor crônica nas costas	nenhum	Desconhecido
8	Feridoun Sabzi^20^	2019	M	76	Desconhecido	válvula sinuso coronariana	Desconhecido	dispneia	nenhum	Recuperação
9	Behnam Shakerian^14^	2019	M	73	16x10mm	septo atrial	Sim	nenhum	nenhum	Recuperação
10	A Angelov^17^	2012	F	28	60x65mm	septo atrial	Não	desconforto no peito, falta de ar e febre	nenhum	Morte

Atualmente, não existe um padrão uniforme para o tratamento de cistos sanguíneos. Portanto, os pacientes com sintomas podem ser acompanhados regularmente. Relata-se que os hemocistos podem causar obstrução do trato de saída ventricular esquerda, disfunção da válvula, disfunção ventricular, derrame embólico, embolia pulmonar e obstrução da artéria coronária.^8^ Portanto, para pacientes com sintomas, deve ser realizado tratamento cirúrgico precoce.

## Conclusão

A ecocardiografia tornou-se a primeira escolha para diagnosticar cistos sanguíneos porque pode nos permitir observar o tamanho, forma, estrutura, função e desenvolvimento valvular do coração, e é não invasiva e segura. Além disso, a ecocardiografia com contraste é útil no diagnóstico de cistos sanguíneos porque pode mostrar se há um preenchimento com meio de contraste na massa. Além disso, a ecocardiografia transesofágica intraoperatória pode ser usada para localizar a massa cardíaca e, assim, ajudar na operação.
