# Supplementary material for: Relato de Caso de Doença Cardíaca Valvular Complicada com um Cisto Sanguíneo no Átrio Direito. Revisão da Literatura
Source: Arq Bras Cardiol. 2021 Nov 22;117(6):1202–6. [Article in Portuguese] doi: 10.36660/abc.20210063 (PMC8757156; doi:10.36660/abc.20210063)
Supplement: Translation

# Translation: A Case Report of Valvular Heart Disease Complicated with a Blood Cyst in Right Atrium. Review of the Literature

**Authors:** Shu Jiang, Xiao-Cong Wang, Yan-Li Zhang, Wei Yu, Li-Ping Pei, Yan Ma

**Affiliations:** 1 Changchun China Jilin University First Hospital, Changchun – China

**Keywords:** Blood Cysts, Cysts/surgery, Heart Valve Diseases, Heart Atria/physiology, Atrial Function, Echocardiography/methods

## Introduction

Blood cysts in the heart are often recognized as benign. They usually do not cause clinical symptoms and are often found in infants younger than two months of age.^1^ In most cases, blood cysts are attached to cardiac valves or their supporting structures.^2^ Cardiac blood cysts in adults are extremely rare, especially in the chambers of the heart. Here, we report a case of valvular heart disease complicated with a blood cyst in the right atrium. Transthoracic echocardiography, contrast echocardiography and esophageal echocardiography were used to examine the patient.

## Case report

The patient was a 64-year-old woman with palpitations and shortness of breath after exercise for four months. In the previous two weeks, the symptoms had worsened. Local hospitals recommended referral to superior hospitals, and then she was referenced to our hospital for treatment. She had a history of hypertension for seven years, smoking for 30 years and hysteromyoma surgery more than 20 years ago. She had no history of diabetes and denied tuberculosis, hepatitis and other infectious diseases. The patient did not have a clear record of medication. At admission, her vital signs were stable. The electrocardiogram showed atrial fibrillation. Systolic murmurs could be heard in the auscultation area of the mitral valve. There were no murmurs in other valve auscultation areas.

Transthoracic echocardiography showed bilateral enlarged atria, mild mitral stenosis and severe insufficiency, with normal left ventricular systolic function (LVEF=52%). In addition, a circular anechoic lesion was approximately 30x41 mm in size in the right atrium, which did not cause tricuspid valve obstruction (Figure 1A).

**Figure 1:** A) Transthoracic echocardiography showed a circular anechoic lesion in the right atrium. B, C) Contrast perfusion imaging showed a cystic structure with a septum and the absence of perfusion in the mass. D) Intraoperative transesophageal echocardiography revealed a circular echo in the right atrium, with spontaneous echocardiographic contrast and septation. RV: right ventricle; LV left ventricle; RA: right atrium; LA: left atrium.

A GE Vivid E9 ultrasound diagnostic device with an M5sC probe was used for a contrast echocardiography examination. SonoVue (1 ml) was slowly injected through the median cubital vein over 1 minute. The scintillation artifact was triggered approximately 2 minutes after injection of the contrast agent. The microbubbles in the myocardium were quickly destroyed when a high MI pulse was emitted. Then the filling process of the contrast agent in the myocardium and tumors was observed in a low-MI state. Contrast perfusion imaging showed a cystic structure with a septum of approximately 36x42 mm in the right atrium and the absence of perfusion in the mass (Figure 1B, C).

Intraoperative transesophageal echocardiography revealed a circular echo in the right atrium, with spontaneous echocardiographic contrast and septation, which we considered to be a blood cyst. (Figure 1D).

The patient underwent right atrial mass excision, mitral valve replacement and tricuspid valvuloplasty. The mass was located in the right atrium connected to the oval fossa, near the orifice of the inferior vena cava, by a pedicle approximately 5 mm in diameter. It was around 40 x 40 mm in size (Figure 2A, 2B). The capsule was intact, tough and purple-black. The atrial septal tissue attached to the tumor and pedicle was completely removed. Mitral valve replacement and tricuspid valvuloplasty were performed.

**Figure 2:** A, B) Surgical findings. The tumor was located in the right atrium connected to the oval fossa, near the orifice of the inferior vena cava, by a pedicle approximately 5 mm in diameter.

Macroscopic examination showed that the right atrial mass was round with a volume of 45x35x27 mm, had a complete capsule, and a narrowed pedicle on the surface. The mass section was unilocular, the cystic wall was 1-2 mm thick, the inner lining was smooth, and the content was mainly coagula. Light microscopic examination of paraffin-embedded sections showed fibrocystic wall-like tissue, interstitial fibrous tissue hyperplasia, hyaline degeneration and mucinous degeneration in the right atrium mass, and massive coagulation tissue, which accorded with the cyst changes (Figure 3A, 3). The valve tissue examined showed hyaline degeneration and mucinous degeneration with scattered infiltration of chronic inflammatory cells (Figure 3C).

**Figure 3:** A, B) Pathology findings. The mass was filled with fibrocystic wall-like tissue, interstitial fibrous tissue hyperplasia, hyaline degeneration and mucinous degeneration in the right atrium mass, and massive coagulation tissue. C: The valve tissue examined showed hyaline degeneration and mucinous degeneration with scattered infiltration of chronic inflammatory cells.

The patient recovered well after the operation. No abnormal mass was found in the right atrium by echocardiography. In addition, her mitral valve prosthesis and tricuspid valve function were normal.

## Discussion

Elsasser first reported blood cysts in 1844.^3^ They are usually of congenital origin and are found within a few months after birth, then disappear spontaneously with time. They mostly occur in cardiac valves, such as mitral valves, tricuspid, aortic, and pulmonary valves. Sometimes they exist in the left atrium and right atrium.^4,5^ Blood cysts in adults are extremely rare, especially in the heart cavity. There are several hypotheses about the causes of hemocyst formation: 1. During the development of valves, blood cysts are formed because the blood is squeezed and gets stuck in the gap that later closes. 2. Possible heteroplastic changes in primary pericardial mesothelial tissue. 3. Sakakibara et al.^6^ suggested that a sudden blockage of circulation causes blood cysts in the atrium or ventricle. They believed that hypoxia, inflammation and bleeding tendencies might be responsible for developing endocardial hematoma into hemocysts. 4. Blood cysts only represent dilated blood vessels. 5. Mucinous degeneration.^7^ However, there is still no consensus on the cause of blood cysts.^4,8,9^ The current patient had mitral insufficiency. Pathological examination of the valve showed hyaline degeneration and mucinous degeneration. It is presumed that this was related to the formation of blood cysts.

Cardiac tumors are mostly benign and myxomatous. They can occur at any age. Myxoma usually occurs on the endocardial surface of the cardiac cavity, 60% -80% of the time in the left atrium. The pedicle attaches to the oval fossa of the atrial septum and has a certain range of motion with the cardiac cycle.

However, myxoma is usually a round or oval strong-echo mass with regular inside echoes. If there is necrosis in the center, it may be an anechoic area.

Left ventricular contrast echocardiography of myxoma shows that the contrast medium is sparse but enhanced in the lesion. Its intensity is lower than that of adjacent myocardial tissue, which is not consistent with this case.

The echocardiographic manifestations of cardiac echinococcosis are cystic space-occupying lesions in the heart. The distribution of cysts in the heart is mainly related to the myocardial blood supply. Therefore, the most common site is the ventricular wall myocardium, which has the most blood supply of the heart, followed by the atrial wall, and the cardiac cavity is less often involved.^10^ However, the patient did not have a clear history of contact with cattle or sheep or an unclean diet, so that diagnosis was not supported.

From 1958 to 2020, there were only ten articles about blood cysts in the right atrium confirmed by pathology. Our review of the English-language literature revealed ten other entries since 1996, describing a total of 10 patients with details of these blood cysts in the right atrium (Table 1). The symptoms of reported blood cysts vary. Four patients were asymptomatic.^11–14^ Other patients had symptoms. The common symptoms were dyspnea and chest discomfort, one case of chronic grief, and one case of headache. The attachment site has also been different. Six cysts were attached to the atrial septum,^11,13-17^ 2 to the oval fossa,^12,18^ 1 to the tricuspid valve,^19^ and 1 to the coronary sinus valve.^20^ Calcification was found in 5 of the blood cysts.^12,14-16,18^ After surgery, most patients recovered,^11-15,18,20^ 2 patients did not have an explained prognosis,^16,19^ and 1 patient died.^17^

**Table 1:** Clinical summary of Blood cysts cases found in English literature since 1958

Patient	First author	Year	Gender	Age	Tumor size	Attachment	Blood cyst with calcium	Symptom	History	Prognosis
1	H Niinam^15^	1996	F	59	20x20mm	atrial septum between the fossa ovalis and tricuspid valve annulus	yes	periodic episodes of substernal pressure and dry cough	none	recovery
2	Hiroyuki Tanaka^11^	2003	M	52	40x30mm	atrial septum between the fossa ovalis and tricuspid valve	no	none	gastric cancer	recovery
3	Gernot Seebacher^18^	2006	F	65	44x20mm	fossa ovalis of atrial septum	yes	dyspnea, angina, and tachycardia	none	recovery
4	Kaoru Otsuka^12^	2007	M	56	30x20mm	fossa ovalis of the atrial septum	yes	none	none	recovery
5	Tomasa Centella^16^	2015	Unknown	62	28x28mm	atrial septum	yes	headache	elevated ferritin unrelated to the hemochromatosis gene, a hiatal hernia, mild esophagitis, and renal sinus cysts	unknown
6	Hiroyuki Otsuka^13^	2016	F	85	30x30mm, 25x25mm	atrial septum between the fossa ovalis and the tricuspid valve	no	none	sick sinus syndrome	recovery
7	Hilary Bews^19^	2018	F	62	45x54mm	tricuspid valve	no	chronic back pain	none	unknown
8	Feridoun Sabzi^20^	2019	M	76	unknown	coronary sinus valve	unknown	dyspnea	none	recovery
9	Behnam Shakerian^14^	2019	M	73	16x10mm	atrial septum	yes	none	none	recovery
10	A Angelov^17^	2012	F	28	60x65mm	atrial septum	no	chest discomfort, shortness of breath, and fever	none	death

At present, there is no uniform standard for the treatment of blood cysts. Therefore, patients with symptoms can be followed up regularly. It is reported that hemocysts may cause left ventricular outflow tract obstruction, valve dysfunction, ventricular dysfunction, embolic stroke, pulmonary embolism and coronary artery obstruction.^8^ Therefore, for patients with symptoms, early surgical treatment should be carried out.

## Conclusion

Echocardiography has become the first choice for diagnosing blood cysts because it can let us observe the size, shape, structure, function and valvular development of the heart, and it is noninvasive and safe. Furthermore, contrast-enhanced echocardiography is helpful in the diagnosis of blood cysts because it can show whether there is a contrast medium filling in the mass. In addition, intraoperative transesophageal echocardiography can be used to locate the cardiac mass and thereby help with the operation.
